# The Macrophage Switch in Obesity Development

**DOI:** 10.3389/fimmu.2015.00637

**Published:** 2016-01-05

**Authors:** Angela Castoldi, Cristiane Naffah de Souza, Niels Olsen Saraiva Câmara, Pedro M. Moraes-Vieira

**Affiliations:** ^1^Department of Immunology, Institute of Biomedical Science, University of São Paulo, São Paulo, Brazil; ^2^Division of Nephrology, Department of Medicine, Federal University of São Paulo, São Paulo, Brazil; ^3^Laboratory of Renal Physiology (LIM 16), Department of Medicine, University of São Paulo, São Paulo, Brazil; ^4^Division of Endocrinology, Diabetes and Metabolism, Beth Israel Deaconess Medical Center, Harvard Medical School, Boston, MA, USA

**Keywords:** obesity, adipose tissue, insulin resistance, macrophage, adipokines, macrophage polarization, adipose tissue inflammation

## Abstract

Immune cell infiltration in (white) adipose tissue (AT) during obesity is associated with the development of insulin resistance. In AT, the main population of leukocytes are macrophages. Macrophages can be classified into two major populations: M1, classically activated macrophages, and M2, alternatively activated macrophages, although recent studies have identified a broad range of macrophage subsets. During obesity, AT M1 macrophage numbers increase and correlate with AT inflammation and insulin resistance. Upon activation, pro-inflammatory M1 macrophages induce aerobic glycolysis. By contrast, in lean humans and mice, the number of M2 macrophages predominates. M2 macrophages secrete anti-inflammatory cytokines and utilize oxidative metabolism to maintain AT homeostasis. Here, we review the immunologic and metabolic functions of AT macrophages and their different facets in obesity and the metabolic syndrome.

## Introduction

Obesity is a prevalent metabolic disease characterized by excess accumulation of white adipose tissue (AT) due to increased food intake and changes in lifestyle (1, 2). Obesity leads to the development of a low-grade systemic chronic inflammatory state (3–6). According to the World Health Organization (WHO), 39% of adults over 18 years of age are overweight and 13% are clinically obese, translating to approximately 2 billion overweight adults where more than half a billion are obese (7).

A major player in systemic low-grade chronic inflammation in obesity is the increased numbers of AT pro-inflammatory macrophages and deregulated production and function of AT hormones and cytokines (2, 4). Besides its role in storing energy, AT is an important endocrine organ (8, 9), such that its dysfunction strongly contributes to the initiation and exacerbation of type 2 diabetes (T2D) (8, 10).

Insulin resistance is defined as a reduced response to insulin in liver, muscle, and AT. This impairment is due to the inhibition of the insulin-signaling pathway, leading to hyperglycemia. Insulin resistance is commonly associated with obesity and may precede the onset of T2D (11–13). One hypothesized reason for impaired insulin signaling has been thought to be due to the chronic systemic low-grade inflammation in obesity (14).

The finding that infiltration of monocytes, which differentiate into macrophages, is augmented in obesity is fundamental (15, 16). This results in pro-inflammatory macrophage and polarization leading to AT inflammation and insulin resistance (15, 17). Importantly, macrophages are crucial for regulating the immune system, specifically by restoring and maintaining AT homeostasis (18, 19).

In this review, we highlight the different functions of AT macrophages (ATMs) in the maintenance AT tissue homeostasis during lean, obese, and insulin resistant states.

## Adipose Tissue Macrophages

The mechanisms by which inflammation increases during obesity are not fully understood. Increased pro-inflammatory cytokine secretion contributes to insulin resistance in obesity. Among these cytokines, tumor necrosis factor-α (TNF-α) was the first cytokine identified to be capable of inducing insulin resistance in adipocytes *in vitro*. In AT, the secretion of TNF-α is primarily derived from macrophages (20–22), and the accumulation of these immune cells in obesity contributes to the development of insulin resistance (23). This supports a key role for inflammation in the regulation of systemic metabolic homeostasis.

Macrophages make up to 40% of all AT cells in obese mice compared to 10% in lean mice (23). These cells are increased in AT during obesity due to increased amounts of several factors, including free fatty acids (FFAs), cholesterol, and lipopolysaccharide (LPS). Serum levels of LPS are elevated in obesity and, this cell wall component from Gram-negative bacteria, is linked to changes in the gut microbiota (metabolic endotoxemia) (24). LPS binds to and activates toll-like receptor 4 (TLR4) and its downstream signaling pathways in AT resident cells. These activated macrophages secrete cytokines and chemokines, such as monocyte chemoattractant protein-1 (MCP-1), and express C–C motif chemokine receptor-2 (CCR2) and CCR5, which in turn augment the recruitment of more monocytes and other leukocytes into AT (25–27). Macrophages share the same differentiation and recruitment molecules with other myeloid cells in many inflammatory conditions (28). As observed during bacterial inflammation (29), in obesity, macrophage activation is dependent on I kappa B kinase-β (IKK-β) (30). Arkan et al. showed that IKK-β activation in macrophages is sufficient for the development of insulin resistance, and mice with loss of IKK-β function only in myeloid cells are protected from obesity development and insulin resistance (30). These findings demonstrate the importance of macrophages in the context of insulin resistance development.

In addition to the activation and inflammatory profile of macrophages in the obese state, ATMs are highly adaptive to its lipid-rich environment. To maintain AT homeostasis in this lipid-rich microenvironment, macrophages increase their adiposity by activating lysosomal lipid metabolism (31). This may be a physiological response to buffer the increase in lipid concentrations released by adipocytes during obesity. This process does not classically activate ATMs, but it activates an immune cell differentiation program where high concentrations of lipids and FFAs induce a macrophage phenotype distinct from differentiated bone marrow macrophages (BMDM) (31). This phenotype is characterized by lipid accumulation in ATMs and increased expression of fatty acids transporters, such as CD36 and the lipid scavenger receptor Msr1 (31).

Several immune cells regulate AT inflammation, insulin resistance (32), and macrophage recruitment and differentiation (19, 33–35). There are two distinct macrophage populations found in AT. In healthy/lean AT, alternatively activated macrophages (M2) that express CD206 and CD301 on their surface and secrete anti-inflammatory cytokines predominates. On the other hand, obesity triggers the accumulation of classically activated macrophages (M1) characterized by CD11c surface expression, and expression of pro-inflammatory cytokines (17, 36), although this pan-classification spans a broad range of macrophage subtypes.

However, Kratz et al. recently described a different subtype of macrophage (37). They observed that treating macrophages with a mix of glucose, palmitate, and insulin (“metabolic activation”) generates a unique macrophage pro-inflammatory phenotype that is different from M1. This type of macrophage secretes pro-inflammatory cytokines, such as interleukin-1β (IL-1β) and TNF-α, whereby the secretion is dependent on peroxisome proliferator-activated receptor gamma (PPAR-γ) and p62 expression. *In vivo*, this phenotype is due to continuous and excessive exposure of ATMs to FFAs, such as palmitate, in a microenvironment that is saturated with glucose and insulin. In obesity, this differentiated macrophage subtype indicates the importance and the necessity to identify differentiated profiles of immune cells. Since there is a large spectrum of ATMs that have different immune profiles, we choose to focus on M1 and M2 subtypes of ATMs to better understand how metabolic alterations in ATMs impact obesity and insulin resistance.

## M1 Macrophages: An Overview

M1 macrophages are associated with a pro-inflammatory profile. These macrophages are generally stimulated by T-helper 1 (Th1) type of cytokines, such as interferon-γ (IFN-γ), or by pathogen-associated molecular patterns (PAMPs), such as LPS (38). In turn, M1 macrophages secrete cytokines, including IL-6, TNF-α, IL-1β (39), IL-12, and IL-23 (40). M1 macrophages can also induce Th1 responses (41, 42). In general, these cells express high levels of major histocompatibility complex class II (MHC-II), CD80 and CD86 costimulatory molecules and CD68 (43). Moreover, M1 macrophages express Th1 cell-attracting chemokines, including CXCL9 and CXCL10 (44).

In addition to IFN-γ and LPS, there are several other molecules involved in M1 macrophage polarization, such as interferon regulatory factor (IRF), signal transducers and activators of transcription (STAT), and suppressor of cytokine signaling (SOCS). IRF5 is involved in M1 polarization by inducing the transcription of interleukin-12 subunit p40 (IL-12p40), IL-12p35, and IL-23p19, and by repressing the transcription of IL-10 (45). M1 macrophages express SOCS3, which promotes nitric oxide (NO) production (46). SOCS3 controls nuclear factor-κB (NF-κB) and phosphatidylinositol 3-kinase (PI3K) activity, favoring NO production in macrophages (46). The induction of inducible nitric oxide synthase (iNOS), another important molecule induced in M1 macrophages is dependent on TLR ligands, such as LPS, and activation of NF-κB, PI3K, and IFN-γ secretion (47, 48) (Table 1). Furthermore, myeloid differentiation primary response gene 88 (MyD88)-dependent pathway is also important for M1 polarization (49). The expression of TLR4/TLR2 is significantly higher in M1 when compared to M2 macrophages (50). The absence of TLR4 drives macrophages toward an M2 phenotype (51), indicating that activation and polarization of macrophages is, at least, in part dependent on TLRs.

**Table 1:** **Differential requirement for stimuli and differential expression of transcription factors, cytokines, chemokines, and other molecules by M1 and M2 macrophages**.

	M1	M2
Classical stimuli	LPS/GM-CSF/IFN-γ/TNF-α	PPAR-γ agonists/IL-4/IL-10/IL-13
Membrane markers	MHCII/CD80/CD86/CD11c/CCR7/Ly6C^high^/CD11b/CD62L/CCR2^high^/CX_3_CR1^low^/CCR5	Dectin-1/CD206/Scavenger receptor/CD163/CCR2^low^/CXCR1/CXCR2/Ly6C^low^/CD11b/CX_3_CR1^high^
Classical transcription factors	STAT1/IRF5	STAT6/FIZZ1/Ym1/PPARα/β/γ
Cytokines and chemokines	IL-6/TNF-α/IL-1β/IL-12/Il-23/IFN-γ/CXCL9,10,11,13/CCL8, 15, 19, 20	TGF-β/IL-10/CCL17, 18, 22, 24
Other classical molecules	SOCS3/iNOS	*Arg1*

In contrast to M1 macrophages generated *in vitro*, which do not express CD11c, M1 ATMs express CD11c concomitant with F4/80 and CD11b (17, 52–54). Interestingly, the expression of CD11c *in vitro* by BMDM can be induced if BMDMs are differentiated in the presence of adipocytes (31, 37). This indicates the importance and requirement of adipocytes in orchestrating the functional phenotype of ATMs.

The recruitment of monocytes, which in AT gives rise to CD11c^+^ ATMs, is dependent on CCR2, CCR5, and MCP-1 (55, 56). Nagareddy et al. demonstrated that ATM-derived IL-1β promotes monocyte release from the bone marrow (57) and MCP-1 induces M1 ATM proliferation in AT (58). These processes are important to promote macrophage accumulation in the AT during obesity and sustain AT inflammation and insulin resistance (58).

## Polarizing M1 ATMs: How They Induce Insulin Resistance

Obesity-associated insulin resistance correlates with elevated levels of pro-inflammatory cytokines, such as TNF-α, IL-1β, and IL-6 (42, 59–62). These cytokines are secreted by both adipocytes and ATMs due to increased levels of pro-inflammatory factors released during obesity development. These factors include FFA, triglycerides, resistin, leptin, retinol-binding protein 4 (RBP4), IL-6, TNF-α, and IL-1β, among others (31, 63, 64).

Secretion of these factors activates several inflammatory signal transduction pathways in macrophages and adipocytes, which are required for obesity-induced insulin resistance. The stress-responsive c-Jun NH_2_-terminal kinase (JNK 1 and 2) (65), inhibitor of κB kinase (IKK) (66), extracellular signal-regulated kinase 1 and 2 (ERK 1 and 2) (67), and mitogen-activated protein kinase p38 (p38 MAPK) are responsible for alterations in the insulin receptor signaling pathway (68). These alterations lead to decreased tyrosine phosphorylation of insulin receptor substrate (IRS-1 and -2), PI3K activation followed by a decreased serine phosphorylation of Akt and consequently insulin resistance (66, 68–72). There is a crosstalk between the two isoforms of JNK (JNK1 and JNK2) that contributes to obesity-induced insulin resistance development. The balance between these two molecules determines the total activity of JNK in fat tissues (73). Hematopoietic activation of JNK1 is a major player in obesity-induced inflammation and insulin resistance (74). Corroborating this, Han et al. verified that knockdown of both JNK 1 and 2 in macrophages protect mice from HFD-induced insulin resistance and AT inflammation (65). Similarly, Vallerie et al. showed that myeloid JNK1 is a regulator of cytokine expression in AT during the late, but not early states of obesity development (75).

Toll-like receptors and inflammasomes are activated in obesity by damage-associated molecular pattern molecules (DAMPs), such as high-mobility group box 1 (HMGB1) and oxidized low-density lipoprotein (Ox-LDL), RBP4 or PAMPs, such as LPS (24, 76–80). TLRs and inflammasomes modulate macrophage polarization due to activation of NF-κB, STAT1, and caspase-1 to induce IL-1β production (81, 82). Upon activation, these receptors contribute to low-grade chronic inflammation in obesity, leading to M1 polarization of ATMs. Importantly, TLR4 expression is increased in ATMs during obesity (83). Thus, many studies have investigated the role of TLR4 and nod-like receptor protein 3 (NLRP3) in knockout mouse models in HFD-induced obesity (17, 23, 51, 84).

Toll-like receptor 4 deficiency in HFD-fed mice ameliorates AT inflammation, insulin resistance, and adiposity (83, 85, 86). The reduction in inflammation is due to decreased macrophage infiltration and a switch from M1 to M2 macrophage profile (51, 83, 85, 87).

Nod-like receptor protein 3 inflammasome also plays a key role in the development of AT inflammation and insulin resistance (88, 89). Expression of NLRP3, apoptosis-associated speck-like protein containing CARD (ASC), caspase-1, and IL-1β are all upregulated in AT of obese mice, as well as the mature form of IL-1β (82, 90). The secreted IL-1β binds to IL-1R and activates NF-κB and MAPK pathways, thereby impairing insulin signaling through the activation of IRS-1 in adipocytes leading to insulin resistance (82, 91).

Functional deletion of NLRP3 and caspase-1 ameliorate HFD-induced insulin resistance and AT inflammation (82, 90, 92). Moreover, weight loss and insulin sensitivity in patients with T2D is associated with decreased AT expression of NLRP3 and IL-1β (82). Protection from insulin resistance and inflammation following loss of functional NLRP3 may be due to a shift in macrophage polarization, since NLRP3-knockout mice have decreased M1 and increased M2 gene expression profiles in AT (84).

In addition to these important signaling pathways, the mammalian target of rapamycin (TOR) has an important function in insulin resistance. It is able to sense nutrients and respond by altering the cellular metabolism in different kind of cells, including ATMs (93). Insulin, glucose, leptin, and other growth factors and cytokines activate mTOR pathway via PI3K–Akt signaling pathway (94). The protein kinase Akt phosphorylates and inhibits TSC2 and, consequently, activates mTORC1 (95, 96). Activation of these metabolic sensors, mainly PI3Kγ, is important for immune cell functions. PI3Kγ activation in hematopoietic cells contributes to the development of obesity and insulin resistance. PI3Kγ activity in the non-hematopoietic compartment is critical during obesity (97). Moreover, the catalytic subunit of PI3Kγ, p110γ, was shown to be activated during obesity. Absence of functional p110 improved insulin sensitivity with reduced infiltration of pro-inflammatory macrophages and inflammatory marker expression in AT. In addition, specific depletion of PI3Kγ in bone marrow cells as well as pharmacological blockade also inhibited macrophage infiltration during obesity and insulin resistance (98). Together, these data indicate that activation of metabolic sensors in immune cells during obesity is essential for inflammation and insulin resistance development.

Defects in mTORC1 regulation can lead to metabolic dysfunction, including T2D (93). Deletion of mTORC1 in macrophages diminishes AT inflammation and protects mice against HFD-induced insulin resistance (99, 100). mTORC1 disruption suppresses HK1-dependent glycolysis, caspase-1 activation, IL-1β, and IL-18 secretion *in vitro* and *in vivo* and induces M2 polarization (100). In accordance, Jiang et al. showed that mTORC1 depletion in macrophages protects mice against HFD-induced AT inflammation and insulin resistance through the inhibition of IRE1α/JNK/NF-κB pathways (99).

In 2013, Horng et al. demonstrated *in vitro* and *in vivo* that TSC1 deletion (Tsc1 deficiency, thereby mTORC1 is constitutively active) in macrophages leads to a marked defect in M2 polarization in response to IL-4, although LPS stimulation induced inflammatory responses in an mTOR-dependent manner (101). Moreover, in obesity, nutrient sensing by mTORC1 regulates the switch of ATMs from M2 to M1 (12).

**Figure 1:**
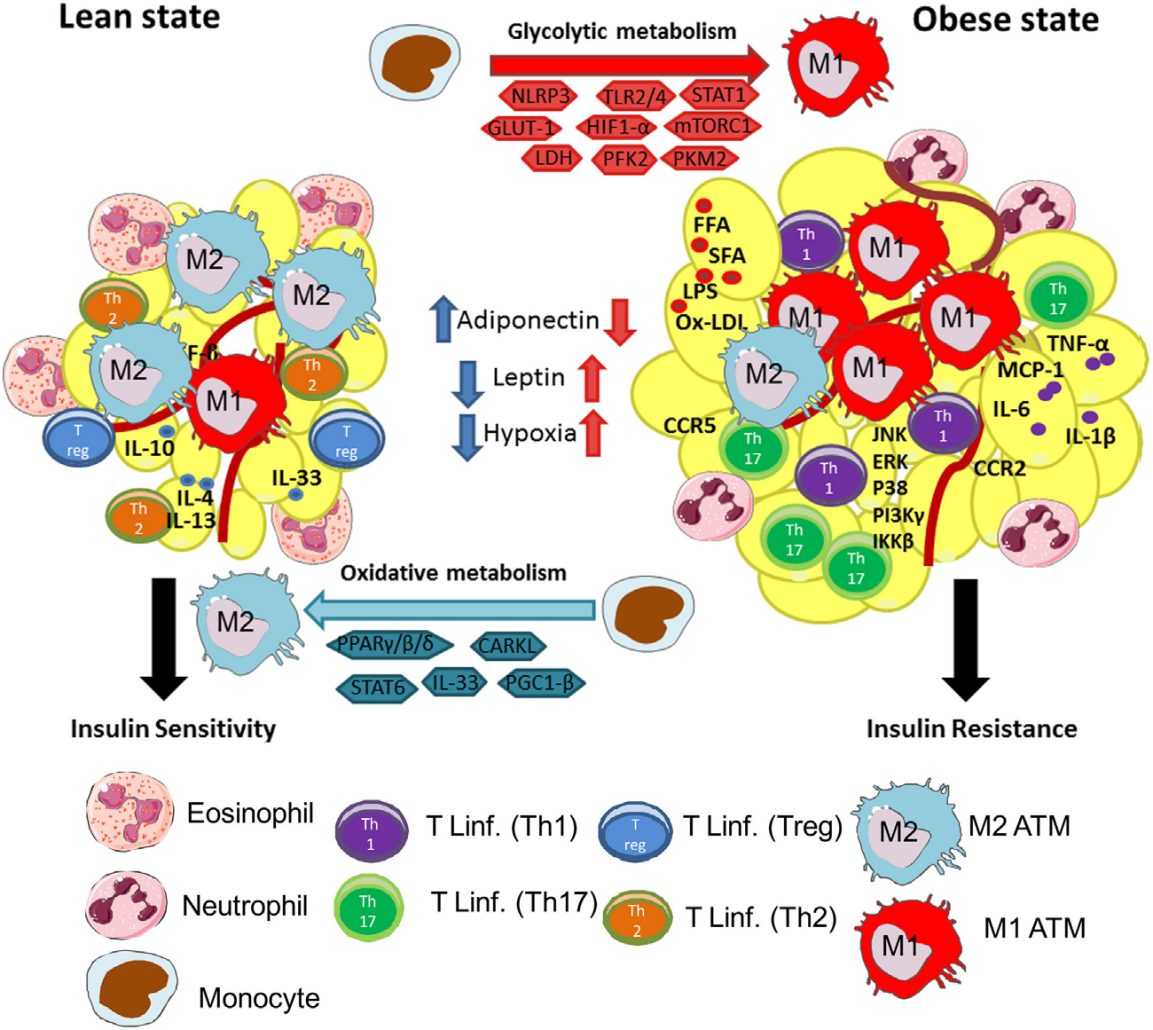
**Macrophages are central players in lean and obese states**. Lean adipose tissue is abundant in immune cells, such as eosinophils, Th2 T cells, ILC2, regulatory T cells (Treg), and M2 (anti-inflammatory) macrophages. These cells are known to secrete anti-inflammatory cytokines, such as IL-10, IL-4, IL-13, and IL-33, to maintain AT homeostasis and controlling insulin sensitivity. M2 macrophages use oxidative metabolism through PPARγ/β/δ, CARKL, STAT6, and PGC-1β. These events are central to maintain a healthy environment in adipose tissue. In the other hand, during obesity, AT is characterized by infiltration of several immune cells, such as monocytes, neutrophils, Th1 and Th17 lymphocytes, and M1 (pro-inflammatory) macrophages. The increased secretion of FFA, SFA, Ox-LDL, and LPS in obesity activates resident macrophages and adipocytes leading to secretion of pro-inflammatory cytokines, such as TNF-α, IL-6, IL-1β, and chemokines MCP-1, CCR2, and CCR5. This process will instigate the recruitment of monocytes and differentiation of M1 macrophages in AT. Besides, activation of pro-inflammatory signaling pathways downstream to TLRs, such as JNK, ERK, p38, IκB, IKKβ, and Pi3Kγ, inhibit insulin receptor signaling, leading to insulin resistance. Moreover, in obese AT, M1 macrophages use glycolytic metabolism and require activation of intracellular molecules, such as NLRP3, TLR2/4, STAT1, GLUT-1, HIF-1α, mTORC1, PFK2, and PKM2, and conversion of pyruvate to lactate by LDH. Activation of glycolysis in macrophages is central to maintain their pro-inflammatory profile.

More recently, Zhu et al. proposed that TSC1 deletion in macrophages intensifies the M1 polarization (102). TSC1 inhibits M1 polarization by suppressing the Ras GTPase/Raf1/MEK/ERK signaling pathway in an mTOR-independent manner, whereas TSC1 promotes M2 properties by mTOR-dependent CCAAT/enhancer-binding protein-β pathway (102). These findings indicate a critical role for TSC1 in orchestrating macrophage polarization via mTOR-dependent and -independent pathways (102) (Figure 1).

Increased M1 activation in AT is involved in activation of the adaptive immune response through the recruitment and activation of T cells. Increased recruitment of CD4^+^ T cells correlates with increased M1 polarization. Also, M1 polarization appears to be dependent on AT Th1 polarization (42, 103–106). In addition, during obesity, the activation of Th1 responses in AT are mediated by mTORC1, since this molecule is necessary for polarization of T lymphocytes toward a Th1 phenotype (107). Moreover, circulating leptin, which is elevated during obesity, activates mTOR pathway, and also induces Th1 responses (108, 109). Thus, Th1 polarization is dependent on M1 polarization, and it is critical for the development of insulin resistance (104).

Together, several pathways mediate the induction/activation of ATMs to maintain AT homeostasis, which can also be affected by changes in systemic and cellular metabolism.

## M2 Macrophage: An Overview

M2 macrophages are associated with tissue remodeling and inflammation resolution (110). M2 macrophages have immunosuppressive properties, have high phagocytic capacity, and secrete extracellular matrix components, angiogenic and chemotactic factors, anti-inflammatory cytokines, and growth factors, such as IL-10 and transforming growth factor β (TGF-β) (111, 112). M2 macrophages are characterized by upregulated expression of Dectin-1, CD206, scavenger receptor A, scavenger receptor B-1, CD163, CCR2, CXCR1, CXCR2, and MgL 1/2 (36). Moreover, the expression of arginase-1 (*Arg1*), PPAR-γ, and transcription factor found in inflammatory zone 1 (FIZZ1), which is specific of murine M2 macrophages, are necessary for collagen synthesis, further supporting the role of these cells in tissue remodeling (44) (Table 1).

*In vitro*, M2 macrophages appear to be a heterogeneous population induced by a variety of stimuli. M2a is induced by IL-4 or IL-13 express high levels of CD206 and has immunoregulatory functions (38, 113–115). M2b is induced by immune complexes and TLRs or IL-1R agonists. Both M2a and M2b have an immunoregulatory role through down-regulation of IL-12, IL-6, and TNF (116). M2c is induced by IL-10 and glucocorticoids. It has an immunosuppressive phenotype and participates in tissue remodeling. M2c secretes pro-fibrotic factors, such as TGF-β, CCL17, and CCL22 (38, 116). In addition, expansion of M2c macrophages is negatively regulated by PPAR-γ, which is expressed in M2 ATM (117). Although significant progress has been made in characterizing M2 subpopulations, it still not completely understood how these cells behave *in vivo*.

## M2 ATMs and Insulin Sensitivity

The microenvironment in a lean AT is composed of a 4:1 M2:M1 ratio (118). The presence of eosinophils and regulatory T cells (Tregs), which secrete the cytokines IL-4/IL-13 and IL-10, respectively, polarizes ATMs toward an anti-inflammatory phenotype (119–121). In lean AT, adipocytes secrete higher levels adiponectin compared with obese AT. Adiponectin enhances insulin sensitivity and increases M2 macrophage polarization (121). These cells and their secretome maintain the positive balance of M2 macrophages in lean AT.

Obesity inversely correlates with AT Tregs (122, 123). Moreover, Tregs can induce M2 macrophage differentiation in mice through IL-10 and TGF-β (124). In lean AT, these cells are involved in the regulation of tissue homeostasis and help to maintain the M2 macrophage population (122).

Recently, new regulatory players in AT homeostasis have been identified: innate lymphoid type 2 cells (ILC2s) and IL-33. ILC2s are a regulatory subtype of ILCs. These cells were divided into three distinct populations, ILCs 1, 2, and 3 (125–127). These subpopulations of ILCs are analogous to the largely known CD4^+^ T helper subsets: Th1, Th2, and Th17, respectively, with respect to cytokine profile expression (128). However, ILCs do not have T-cell receptors and respond to antigenic signals in the absence of antigen specificity (128). ILCs are activated by the cytokine IL-33 and produce large amounts of the type 2 cytokines: IL-5 and IL-13 (129).

Interleukin-33 is constitutively present in humans and mice, mainly in specialized populations of epithelial and endothelial cells (130, 131). Its receptor (ST2) is highly expressed in ILC2s and Th2 lymphocytes, and it is also found in eosinophils, mast cells, dendritic cells, basophils, myeloid-derived suppressor cells, and Tregs (132).

Interleukin-33, as well as ILC2s, has been in the spotlight due to their putative contributions in the improvement of obesity-induced insulin resistance. Upon binding to its receptor, IL-33 induces the production of large amounts of anti-inflammatory cytokines by AT ILC2s and also the polarization of ATMs toward an M2 phenotype (133). This results in AT mass reduction and improves insulin resistance (133, 134). Han and colleagues investigated ST2 expression in murine Tregs in lean and obese visceral AT. AT Tregs from lean mice express higher levels of ST2 compared to AT Tregs from obese mice. Moreover, treatment with IL-33 restored the ST2-positive Treg population, reduced AT inflammation, and improved insulin resistance (133).

In this context, Brestoff et al. demonstrated that IL-33 plays an important role in the maintenance of ILC2s in AT, promoting energy expenditure, and reducing adiposity in mice (135). This decrease in adiposity was due to caloric expenditure upon the induction of uncoupling protein 1 (UCP1) expression in AT, a process called “beiging” or “browning” (136, 137). UCP1 protein is limited to beige and brown adipocytes and regulates caloric expenditure (135). In agreement with Artis et al., Chalwa’s et al. found that IL-33 promoted the accumulation and activation of ILC2s in mouse AT, leading to the biogenesis of beige fat, which is crucial for AT metabolic homeostasis (138) (Figure 1).

Taken together, these studies demonstrate the importance of alternatively activated macrophages to maintain the tissue homeostasis, especially in AT. Moreover, the discovery of new alternative pathways for the polarization of ATMs toward an M2 phenotype is necessary to better understand the mechanisms by which insulin sensitivity in obesity.

## Macrophage Metabolism and Its Role in Insulin Sensitivity

In addition to cytokines, the availability of substrates in tissues orchestrates macrophage function. Cellular metabolism is not static but is rather a dynamic process that allows cells to adapt to the microenvironment (139). The type of nutrient substrate is critical for ATM function. Saturated fatty acids (SFAs) are pro-inflammatory and induce M1-like phenotype, while certain types of unsaturated fatty acids (UFAs), such as omega-3 and branched fatty acid esters of hydroxy fatty acids (FAHFA) (140), are anti-inflammatory and induce an M2-like phenotype (141).

M1 macrophages preferentially metabolize glucose as an energy substrate (142). During activation, macrophages alter its metabolism to support survival and cellular functions. The metabolism of M1 macrophages upon activation is characterized by induced aerobic glycolysis with increased glucose uptake and the conversion of pyruvate to lactate by lactate dehydrogenase (LDH) (143). This activation in aerobic glycolysis decreases respiratory chain activity due to increased ROS levels (144). This metabolic switch is necessary for NO production, an important effector of immune microbicidal activity and pro-inflammatory M1 macrophage responses (144).

In addition, the expression of glucose transporter-1 (GLUT-1) drives the pro-inflammatory phenotype of M1 macrophages, increases glucose uptake, and, subsequently, augments glucose metabolism (145).

One important molecule regulating glycolysis and macrophage activation is hypoxia inducible factor-1α (Hif-1α). Hif-1α induces a pro-inflammatory phenotype in macrophages (146) via TLR4 activation, which involves the PI3K/Akt signaling pathway (147). Low oxygen (O_2_) tension and inflammatory responses increase TLR4 expression in macrophages (148). Moreover, M1 macrophages co-localize with AT hypoxic areas in obese mice and are associated with increased inflammatory responses (147–149). Because these macrophages need to adapt to the obesity-induced hypoxic tissue environment, activating anaerobic glycolysis under these circumstances best serves these immune cells to support their rapid and demanding energy requirements (143).

Activation of macrophages with LPS also results in increased levels of succinate and malate (150). Succinate, in particular, drives IL-1β production, which is dependent on Hif-1α activation (150). In addition, pyruvate kinase M2 (PKM2), a critical determinant of macrophage activation by LPS, promotes inflammatory responses (151). Activation of PKM2 plays a key role in stabilizing Hif-1α and Hif-1α-dependent genes, such as IL-1β expression. LPS induces dimerization of PKM2 that in turn complexes with Hif-1α. This complex directly binds to the IL-1β promoter, an event that is inhibited by the activation of tetrameric PKM2, which induces M2 macrophage differentiation and attenuates LPS-induced M1 macrophages (151). Thus, PKM2 in its dimeric form is required for glycolytic reprograming in response to LPS. The dimeric form of PKM2 plays role in Hif-1α function, whereas the tetrameric form of PKM2 impairs the ability of PKM2 to promote transcriptional activity of Hif-1α and LPS-induced IL-1β expression (151).

Nonetheless, the microenvironment rich in LPS and IFN-γ also enhances M1 macrophage polarization and glycolysis activation independently of Hif-1α. This occurs upon 6-phosphofructo-2-kinase/fructose-2,6-bisphosphatase (PFK2) induction (152).

In contrast to M1 glycolytic metabolism, M2 macrophages utilize oxidative metabolism (142). The induction of oxidative metabolism in M1 macrophages shifts their phenotype toward an M2 profile (152). Moreover, the overexpression of carbohydrate kinase-like protein (CARKL), which regulates the production of sedoheptulose-7-phosphate (S7P), an intermediate of the pentose phosphate pathway (PPP) (153) results in decreased production of pro-inflammatory cytokines, which suggests a shift toward an M2 macrophages phenotype (154).

Besides CARKL, the coactivator protein PPAR-γ-coactivator-1β (PGC1-β) induces mitochondrial respiration as well as mitochondrial biogenesis. This is a key player in the metabolic switch of macrophages from M1 to M2 phenotype (142, 144). Blocking PGC1-β results in impaired M2 macrophage metabolism and function (142). Thus, identifying mechanisms that modulate the metabolism of macrophages may dampen the onset and exacerbation of inflammatory processes.

Adipose tissue-derived IL-4 and IL-13 signals through IRF/STAT to activate STAT6 in M2 macrophages (44, 113). STAT6 induces the expression of transcriptional regulators, such as PPAR-γ (44). PPAR-γ maintains the metabolic switch toward oxidative metabolism and promotes M2 gene expression (*Arg1*) to amplify the effector phenotype of M2 macrophages (collagen synthesis) (31, 155, 156). Other members of the PPAR family, PPARβ/δ, appears to differentially influence macrophage activation, along with IL-4 and IL-13, and promotes an alternative M2 macrophage phenotype (156). Myeloid deletion of PPARβ/δ leads to glucose intolerance and insulin resistance (27), indicating that expression of PPARs transcription factors is crucial to maintain the M2 phenotype through the secretion of Th2 cytokines (Figure 1).

Hypoxia inducible factor-2α has been shown to regulate the transcription of *Arg1*, which is expressed by M2 macrophages (157). However, Hif-2α also controls IL-1β production and NF-κB activity, which is associated with an M1 phenotype (150, 157). Thus, although Hif-2α appears to have a role in macrophage polarization, more studies are needed to better understand the importance of this transcription factor for macrophage phenotype, metabolism, and function.

It is still unclear how M2 macrophages metabolism is regulated during obesity and the role of M2 macrophage metabolism for the development of insulin resistance. Nevertheless, in lean state, they have an oxidative metabolism, which may shift to glycolytic metabolism, during obesity, due to a pro-inflammatory environment and further studies are needed to better understand their role in obesity.

## Conclusion

Macrophages are central mediators of obesity-induced AT inflammation and insulin resistance. They also are key cells for maintenance of AT homeostasis. Recently, several reports described the importance of these cells as regulators of insulin sensitivity, which involves the activation of innate immune receptors, transcription factors, and intracellular metabolism to support the either pro- or anti-inflammatory AT phenotype. Thus, macrophages have a dual role, changing their status to support immune responses, obesity development, and related diseases.

## Author Contributions

AC and CN have contributed equally in the writing of this manuscript. NC and PM-V contributed in the writing and corrections of this manuscript. AC and CN have contributed equally to this review.

## Conflict of Interest Statement

The authors declare that the research was conducted in the absence of any commercial or financial relationships that could be construed as a potential conflict of interest.
